# Theory, Analysis, and Applications of the Entropic Lattice Boltzmann Model for Compressible Flows

**DOI:** 10.3390/e22030370

**Published:** 2020-03-24

**Authors:** Nicolò Frapolli, Shyam Chikatamarla, Ilya Karlin

**Affiliations:** Department of Mechanical and Process Engineering, ETH Zurich, CH-8092 Zurich, Switzerland

**Keywords:** entropy, lattice Boltzmann method, compressible flow, nonlinear acoustics, shock waves

## Abstract

The entropic lattice Boltzmann method for the simulation of compressible flows is studied in detail and new opportunities for extending operating range are explored. We address limitations on the maximum Mach number and temperature range allowed for a given lattice. Solutions to both these problems are presented by modifying the original lattices without increasing the number of discrete velocities and without altering the numerical algorithm. In order to increase the Mach number, we employ shifted lattices while the magnitude of lattice speeds is increased in order to extend the temperature range. Accuracy and efficiency of the shifted lattices are demonstrated with simulations of the supersonic flow field around a diamond-shaped and NACA0012 airfoil, the subsonic, transonic, and supersonic flow field around the Busemann biplane, and the interaction of vortices with a planar shock wave. For the lattices with extended temperature range, the model is validated with the simulation of the Richtmyer–Meshkov instability. We also discuss some key ideas of how to reduce the number of discrete speeds in three-dimensional simulations by pruning of the higher-order lattices, and introduce a new construction of the corresponding guided equilibrium by entropy minimization.

## 1. Introduction

Predicting behavior of compressible flows is crucial in a variety of natural phenomena and technological applications, ranging from magnetohydrodynamics and astrophysics to high speed aerodynamic, turbomachinery, and abrasive blasting, to mention a few. While compressible flows are well established by the Navier–Stokes and energy equations, computations remain difficult in practice [1,2]. Particularly challenging is the interaction of shock waves with turbulence which requires special treatment by conventional flow solvers. Indeed, higher-order discretization methods used for the direct numerical simulation (DNS) of turbulent flows can cause Gibbs oscillations, leading to instabilities in the presence of shocks, whereas typical methods used to regularize shock computations demonstrate numerical dissipation which degrades accuracy of DNS in regions of high turbulence [2]. Therefore, hybrid schemes combining higher-order methods with (W)ENO schemes through state-of-the-art shock sensors is the preferred approach to the simulation of high-speed compressible flows. The main drawback of these approaches is the complexity of resulting numerical schemes, requiring flow-specific parameter tuning. Thus, development of efficient, high-fidelity, robust DNS solvers which can treat seamlessly a wide range of compressible turbulent sub-, trans- and supersonic flow is a topic of paramount importance.

The lattice Boltzmann method (LBM) gained attention as an alternative for complex flows simulations including turbulence, micro-scale flows, porous media, multiphase flows, and beyond [3]. In LBM, one solves numerically a set of fully discrete (in time-space-velocity) kinetic equations for the populations $f_{i}\left(x,t\right)$, designed to recover the target equations of fluid dynamics in the hydrodynamic limit. Populations are associated with discrete velocities $v_{i}$ which fit into a regular spatial lattice with nodes x. LBM is realized by an explicit stream-along-links-and-equilibrate-at-nodes algorithm which achieves both computational efficiency and numerical accuracy. Moreover, the entropic version of LBM (ELBM) for incompressible flows [4,5,6] enabled high Reynolds number simulations by restoring the second law of thermodynamics (Boltzmann’s *H*-theorem). This basic physical requirement rendered the method nonlinearly stable numerically.

Extension of LBM to compressible flows requires lattices with more discrete velocities (multi-speed or higher-order lattices) in order to adequately represent the moment system necessary to recover the compressible Fourier–Navier–Stokes equations. Early attempts to introduce higher-order lattices were based on a discretization the Maxwell–Boltzmann distribution on the roots of Hermite polynomials [7]. While this route of higher-order Gauss–Hermite quadratures promised a systematic derivation of new complete Galilean-invariant LB models, the corresponding discrete velocities cannot be fitted into a regular space-filling lattice. Thus, one of the most important advantages of the LB methods, the exact space discretization of the advection step, is lost with the Gauss–Hermite quadrature-based off-lattice models. Regular space-filling lattices were obtained based on a quadrature representation of the equilibrium moments not necessarily Gauss–Hermite [8,9,10,11], where the nodes of the quadrature are chosen empirically in such a way as to reflect higher-order isotropy. However, none of these derivations so far could deliver an accurate numerical scheme without employing some stabilization techniques in the simulation of compressible flows [12].

An alternative way of deriving space-filling higher-order lattices was set by the development of the theory of admissible higher-order lattices [13,14]. This enabled a systematic derivation of lattices supported by the entropy function and thus providing for ELBM realization. First, application of admissible higher-order lattices to turbulent flows simulations was demonstrated in [15].

This paper reviews and extends the results by the authors for compressible ELBM on higher-order lattices [16,17,18,19] and includes new findings first presented in the Doctoral Thesis of the first author in 2017 [20]. We review a generic lattice Boltzmann model for polyatomic molecules which enables variable specific heat and Prandtl number, together with a brief reminder of the theory of admissible lattices [13,14]. While our previous publications have already used the construction of the corresponding lattice equilibria by a direct numerical minimization of the entropy function, here we discuss at length the advantages of this method over the conventional polynomial approximation. We address restrictions on the maximum Mach number and the temperature range allowed for a given lattice. Extension of the operation domain in terms of Mach number and temperature range is presented by modifying the original lattices without increasing the number of discrete velocities and without modification of the numerical algorithm. In order to increase the Mach number, we employ shifted lattices while the magnitude of lattice speeds is increased in order to extend the temperature range. Accuracy and efficiency of the shifted lattices are demonstrated with simulations of the supersonic flow field around a diamond-shaped and NACA0012 airfoil, the subsonic, transonic and supersonic flow field around the Busemann biplane, and the interaction of vortices with a planar shock wave. For the lattices with extended temperature range, the model is validated with the simulation of the Richtmyer–Meshkov instability. We also demonstrate application to an engineering-relevant flow over the Onera M6 wing. We conclude the paper with a discussion of reducing the number of lattice speeds by pruning and guided entropic equilibrium construction first presented in the Doctoral Thesis of the first author in 2017 [20].

## 2. Compressible Lattice Boltzmann Models

### 2.1. Discrete Kinetic Equations

With the compressible entropic lattice Boltzmann model, we solve two sets of kinetic equations for the populations $f_{i}$ and $g_{i}$:

(1) $$\begin{array}{c} f_{i}\left(x+v_{i},t+1\right)-f_{i}\left(x,t\right)=\Omega_{i}^{f}, \end{array}$$

(2) $$\begin{array}{c} g_{i}\left(x+v_{i},t+1\right)-g_{i}\left(x,t\right)=\Omega_{i}^{g}. \end{array}$$

The locally conserved fields, the mass density ρ, the momentum density ρu, where u is the flow velocity, and the total energy density ρE are assembled with the help of the populations *f* and *g* as follows:

(3) $$\begin{array}{cc} \rho & =\sum_{i=1}^{n}f_{i}, \end{array}$$

(4) $$\begin{array}{cc} \rho u & =\sum_{i=1}^{n}f_{i}v_{i}, \end{array}$$

(5) $$\begin{array}{cc} 2\rho E & =2\rho C_{v}T+\rho u^{2}=\sum_{i=1}^{n}f_{i}v_{i}^{2}+\sum_{i=1}^{n}g_{i}. \end{array}$$

Here, *T* is the temperature and $C_{v}$ is specific heat at constant volume which we assume to be independent of the temperature below.

For a *D*-dimensional monatomic ideal gas, the specific heat is $C_{v}=D/2$ (gas constant R=1 in the following) and the adiabatic exponent becomes fixed to $\gamma =\frac{D+2}{D}$. In this case, the first Equation (1) suffices and can be employed to recover also the temperature equation by satisfying conservation of the total kinetic energy,

(6) $$2\rho K=\rho DT+\rho u^{2}=\sum_{i=1}^{n}f_{i}v_{i}^{2}.$$

For polyatomic gases, in order to overcome the fixed adiabatic exponent constraint arising from Equation (1) alone, several approaches have been proposed in the literature [21,22]; however, they are limited to models with a prescribed closed-form expression of the equilibrium. As it will be explained below, the equilibrium employed in the present model does not offer a closed-form solution; therefore, we employ an alternative way, which follows the idea of Rykov’s kinetic model for diatomic molecules [23] and which was already presented for the lattice Boltzmann method in [24]. To that end, a second set of populations $g_{i}$ Equation (2) is introduced; these populations carry the energy related to internal degrees of freedom (rotational and vibrational) and thus enables a variable γ. It is interesting to note that the two-population approach in LBM is similar to the rational extended thermodynamics modeling of polyatomic gases [25].

The left-hand side of Equations (1) and (2) is the standard lattice Boltzmann shift operator while the right-hand side is the collision operator representing the relaxation of the populations to the equilibrium. The collision operator $\Omega_{i}^{f}$ is given by the quasi-equilibrium model [26] combined with the entropic collision [6,13]:

(7) $$\Omega_{i}^{f}=\alpha \beta_{1}\left(f_{i}^{\mathrm{eq}}-f_{i}\right)+2\left(\beta_{1}-\beta_{2}\right)\left[f_{i}^{*}-f_{i}^{\mathrm{eq}}\right].$$

The first term in Equation (7) becomes the conventional LBGK model with α=2 while it represents the entropic collision when α is computed by satisfying the discrete-time *H*-theorem, i.e., when it is computed as the positive root of the entropy condition:

(8) $$H\left(f+\alpha \left(f^{\mathrm{eq}}-f\right)\right)=H\left(f\right)$$

where *H* is the entropy function [4],

(9) $$H\left(f\right)=\sum_{i=1}^{n}f_{i}\ln\left(\frac{f_{i}}{W_{i}}\right).$$

For more details about the entropic estimate in the context of the compressible model, the reader is referred to [18].

The second term in Equation (7) represents the relaxation to a quasi-equilibrium state and is employed to modify the Prandtl number [18]. The collision operator for the second set of populations $g_{i}$ is designed to be consistent with the $f_{i}$ equations and reads:

(10) $$\Omega_{i}^{g}=\alpha \beta_{1}\left(g_{i}^{\mathrm{eq}}-g_{i}\right)+2\left(\beta_{1}-\beta_{2}\right)\left[g_{i}^{*}-g_{i}^{\mathrm{eq}}\right].$$

Note that the equilibrium $g_{i}^{\mathrm{eq}}$ is dictated by $f_{i}^{\mathrm{eq}}$,

(11) $$g_{i}^{\mathrm{eq}}=\left(C_{v}-\frac{D}{2}\right)Tf_{i}^{\mathrm{eq}}.$$

In both collision operators, $f_{i}^{\mathrm{eq}}$ denotes equilibrium states while $f_{i}^{*}$ is the quasi-equilibrium state. Since equilibrium is a central point in the construction of the compressible model, we shall provide some details of it in a separate Section 2.4. The quasi-equilibrium states need to be chosen depending on the Prandtl number [16], and, in particular, for the case Pr≤1, the quasi-equilibrium state conserves the tensor of centered third-order moments $\bar{Q}$. It can be written in the compact form as:

(12) $$f_{i}^{*}=f_{i}^{\mathrm{eq}}+W_{i}\frac{\left(\bar{Q}-Q^{\mathrm{eq}}\right):\left[v_{i}⊗v_{i}⊗v_{i}-3Tv_{i}I\right]}{6T^{3}},$$

where

(13) $$\bar{Q}=\sum_{i=1}^{n}f_{i}\left(v_{i}-u\right)⊗\left(v_{i}-u\right)⊗\left(v_{i}-u\right).$$

This is similar to the Shakhov’s S-model [27]; the difference is that the full third-order moment tensor is used in the quasi-equilibrium (12) rather than the once-contracted form thereof as in [27], cf. [18]. Quasi-equilibrium populations $g_{i}^{*}$ are chosen consistently with the populations $f_{i}^{*}$ and read:

(14) $$g_{i}^{*}=g_{i}^{\mathrm{eq}}+\frac{W_{i}\left(\bar{q}-q^{\mathrm{eq}}\right)\cdot v_{i}}{T},$$

where $\bar{q}$ is the energy flux associated with the internal degrees of freedom of the polyatomic gas:

(15) $$\bar{q}=\sum_{i=1}^{n}g_{i}\left(v_{i}-u\right).$$

In the above expressions, $W_{i}=W_{i}\left(T\right)$ are the lattice-dependent weights that will be detailed later. While the present model is generic in nature, the construction of the equilibrium and the choice of the lattice still need to be specified.

### 2.2. Thermo-Hydrodynamic Equations

Provided that the discrete velocity set is sufficiently isotropic [18], the above kinetic systems (1) and (2) recover, in the hydrodynamic limit, the continuity, the momentum, and the temperature equations as follows (detailed derivation is given in [18]):

(16) $$\begin{array}{cc} \; & \partial_{t}\rho +\nabla \cdot \left(\rho u\right)=0, \end{array}$$

(17) $$\begin{array}{cc} \; & \partial_{t}u+u\cdot \nabla u+\frac{1}{\rho }\nabla \left(\rho T\right)+\frac{1}{\rho }\nabla \cdot \Pi^{\mathrm{neq}}=0, \end{array}$$

(18) $$\begin{array}{cc} \; & \partial_{t}T+u\cdot \nabla T+\frac{T}{C_{v}}\nabla \cdot u+\frac{1}{\rho C_{v}}\nabla u:\Pi^{\mathrm{neq}}+\frac{1}{\rho C_{v}}\nabla \cdot q^{\mathrm{eq}}=0. \end{array}$$

Here, the nonequilibrium stress tensor is defined as

(19) $$\Pi^{\mathrm{neq}}=-\mu \left(S-\frac{2}{D}\left(\nabla \cdot u\right)I\right)+\xi \left(\nabla \cdot u\right)I,$$

with the strain rate tensor,

(20) $$S=\nabla u+\nabla u^{†}.$$

Furthermore, the nonequilibrium heat flux follows the Fourier law:

(21) $$q^{\mathrm{neq}}=-\kappa \nabla T.$$

Transport coefficients, the dynamic viscosity μ, the bulk viscosity ξ, and the thermal conductivity κ are

(22) $$\begin{array}{cc} \mu & =\left(\frac{1}{2\beta_{1}}-\frac{1}{2}\right)\rho T, \end{array}$$

(23) $$\begin{array}{cc} \kappa & =C_{p}\left(\frac{1}{2\beta_{2}}-\frac{1}{2}\right)\rho T, \end{array}$$

(24) $$\begin{array}{cc} \xi & =\left(\frac{1}{C_{v}}-\frac{2}{D}\right)\mu , \end{array}$$

while the adiabatic exponent γ reads

(25) $$\begin{array}{cc} \gamma & =\frac{C_{p}}{C_{v}},\;C_{p}=C_{v}+1. \end{array}$$

Thus, the Prandtl number is expressed in terms of the two parameters of the model, $\beta_{1}$ and $\beta_{2}$ as follows:

(26) $$\begin{array}{c} \mathrm{Pr}=\frac{\left(1-\beta_{1}\right)\beta_{2}}{\left(1-\beta_{2}\right)\beta_{1}}. \end{array}$$

### 2.3. Lattices

In order to simulate thermal or fully compressible flows, we here employ higher-order lattices. We consider those emerging from the theory of admissible higher-order lattices, presented for the first time in [13,14,28]. The construction of the admissible lattices in any space dimension begins with identifying the one-dimensional velocity sets $V_{n}$. A weight $w_{i}\left(T\right)>0$ corresponds to each discrete velocity $v_{i}\in V_{n}$ provided the temperature is within a range $T\in [T_{min},T_{max}]$. Outside the temperature range, the weights and hence the equilibrium populations become negative and the scheme may become numerically unstable. Therefore, the ratio $T_{max}/T_{min}$ is an important characteristic to determine which lattice can be employed for the problem at hand. A summary is provided in Table 1 for one-dimensional admissible velocity set up to n=11. Table 1 shows, for each one-dimensional discrete velocity set $V_{n}$, the temperature range, the reference temperature $T_{0}$, the reduced temperature $\theta =T/T_{0}-1$ range, and the ratio between the maximal and the minimal temperature $T_{max}/T_{min}$. Another restriction is the deviation of the third- and fourth-order equilibrium moments from their Maxwell–Boltzmann (continuous) counterparts. As the number of velocities is increased, for a given Mach number, the errors in the recovering of the Maxwell–Boltzmann equilibrium moments are reduced, as it will be shown below in Section 2.4.

In higher dimensions, the weight $W_{i}$ of each discrete velocity $v_{i}$ in the natural Cartesian reference frame, $v_{i}=\left(v_{ix},v_{iy},v_{iz}\right)$, is the algebraic product of the corresponding one-dimensional weights,

(27) $$W_{i}=w_{ix}w_{iy}w_{iz}.$$

It has been shown elsewhere [17,18] that, in order to simulate compressible flows, the minimum number of velocities is n=7. With this lattice, the errors in the third-and fourth-order moments are kept small up to a moderately supersonic Mach number and the temperature range is sufficient to guarantee positivity of the populations for moderate temperature jumps across shock waves. Therefore, in the remainder of this paper, the reference lattice for compressible flows will be *DdQ*$7^{d}$, D=d is the space dimension; the lattice is constructed by the tensor product of *D* one-dimensional velocity sets $V_{7}$. For this lattice, the one-dimensional weights read:

(28) $$\begin{array}{cc} w_{0} & =\frac{36-49T+42T^{2}-15T^{3}}{36},\\ w_{\pm 1} & =\frac{12T-13T^{2}+5T^{3}}{16},\\ w_{\pm 2} & =\frac{-3T+10T^{2}-5T^{3}}{40},\\ w_{\pm 3} & =\frac{4T-15T^{2}+15T^{3}}{720}. \end{array}$$

**Figure 1:**
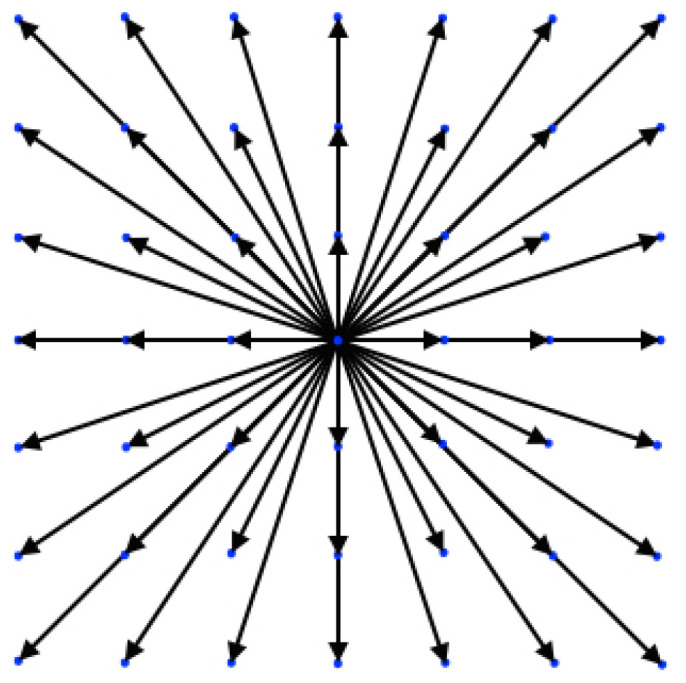
Visualization of the *D*2*Q*49 lattice.

In Figure 1, the *DdQ*$7^{d}$ lattice is shown for the two-dimensional case, d=2.

### 2.4. Equilibrium

The equilibrium computation is one of the key elements of the compressible entropic lattice Boltzmann model. We start by presenting the equilibrium derivation based on the entropy function minimization. Next, we access the computational cost of the accurate equilibrium evaluation and make a comparison with other types of approximation of the equilibrium. We proceed by detailing the accuracy, in terms of Mach number and temperature that can be achieved with the present approach. We conclude this section with further details about the equilibrium by presenting related properties such as positivity of the entropic equilibrium.

#### 2.4.1. Equilibrium Construction

We proceed with the definition of the equilibrium which is derived by minimization of the entropy function *H* (9) under constraints of local conservation of mass ρ, momentum ρu and kinetic energy *K*:

(29) $$\left\{\rho ,\rho u,2\rho K\right\}=\left\{\rho ,\rho u,D\rho T+\rho u^{2}\right\}=\sum_{i=1}^{n}\left\{1,v_{i},v_{i}^{2}\right\}f_{i}\left(\rho ,u,T\right).$$

The minimization problem is solved with the method of the Lagrange multipliers (LM), leading to

(30) $$f_{i}^{\mathrm{eq}}=\rho W_{i}\exp\left(\chi +\zeta \cdot v_{i}+\lambda v_{i}^{2}\right),$$

where χ(u,T), ζ(u,T) and λ(u,T) are Lagrange multipliers corresponding to conservation of mass, momentum and kinetic energy, respectively. The closed form of $f_{i}^{\mathrm{eq}}$ is computed by applying the conservation laws (29) to the equilibrium populations (30). This results in D+2 equations for D+2 unknown LM. For the standard lattice ($v_{i}=\left\{0,\pm 1\right\}$), this problem was solved analytically in [6]. However, for higher-order lattices, subject to energy conservation, this leads to a system of higher-order algebraic equations to which one fails to find a closed-form solution.

We suggest a direct numerical evaluation of the Lagrange multipliers using rapidly converging Newton–Raphson iterations [17]. Our simulations shows that such an approach converges to an accurate solution (with the error of the order $∼10^{-14}$) within five iterations when Lagrange multipliers from the previous time step are used as the initial guess for the Newton–Raphson solver. At first glance, the proposed evaluation of the equilibrium at every node and every time step appears computationally demanding. In the following, we analyze how the numerical computation of the equilibrium impacts the overall computational load of the algorithm.

#### 2.4.2. Computational Costs of Numerical Equilibrium

In order to estimate performance of the accurate numerical equilibrium, and to compare it to the standard polynomial equilibrium evaluation, we report here the computational time required to run a constant homogeneous advection flow at Mach number ${\mathrm{Ma}}_{a}=0.05$. It must be noted that these computational times are evaluated for the full implementation (advection and collision) of the LBGK algorithm with periodic boundary conditions. We consider the accurate numerical equilibrium (30), and a fourth-order polynomial approximation thereof. The fourth-order approximation is theoretically applicable only if the number of the discrete velocities is large enough, i.e., if the lattice velocity space can accommodate all higher order moments required for recovering the compressible flow hydrodynamic equations. The fourth order approximation is given by:

(31) $$f_{i}^{\mathrm{eq}}=\rho W_{i}\left(1+\frac{u_{\alpha }v_{i\alpha }}{T}+\frac{u_{\alpha }u_{\beta }X_{i\alpha \beta }}{2T^{2}}+\frac{u_{\alpha }u_{\beta }u_{\gamma }Y_{i\alpha \beta \gamma }}{6T^{3}}+\frac{u_{\alpha }u_{\beta }u_{\gamma }u_{\mu }Z_{i\alpha \beta \gamma \mu }}{24T^{4}}\right),$$

with $X_{i\alpha \beta }=v_{i\alpha }v_{i\beta }-T\delta_{\alpha \beta }$, $Y_{i\alpha \beta \gamma }=X_{i\alpha \beta }v_{i\gamma }-v_{i\alpha }T\delta_{\beta \gamma }-v_{i\beta }T\delta_{\alpha \gamma }$, and $Z_{i\alpha \beta \gamma \mu }=Y_{i\alpha \beta \gamma }v_{i\mu }-X_{i\alpha \beta }\delta_{\gamma \mu }T-X_{i\alpha \gamma }\delta_{\beta \mu }T-X_{i\beta \gamma }\delta_{\alpha \mu }T$. Note that the polynomial equilibrium (31) can only be used for the simulation of low Mach number flows due to truncation errors and loss of positivity of the populations at higher Mach numbers, as it will be discussed in more detail in Section 2.4.4. In Table 2, we report the computational time normalized by the fastest evaluation, namely the polynomial evaluation on the *DdQ*$3^{d}$ lattice, for the two different equilibrium evaluations, for three lattices, *DdQ*$3^{d}$, *DdQ*$5^{d}$, and *DdQ*$7^{d}$, in two and three dimensions.

It is interesting to notice that the computational time required by the numerical equilibrium on every lattice is not more than twice the computational time of the polynomial equilibrium. Once the number of speeds is increased, the difference between the numerical equilibrium and the polynomial form decreases, in both two and three dimensions. This is explained by the fact that numerical equilibrium computes D+2 Lagrange multipliers, independently of the number of discrete speeds *n*, while, for the polynomial form, the *n* fourth-order polynomials need to be computed. Table 2 suggests that the computational cost depends mainly on the number of discrete lattice velocities, while the type of the equilibrium plays a secondary role. This analysis shows that the use of the accurate form of the equilibrium is not heavily penalizing the performance of the simulation relative to a polynomial form of the equilibrium.

A general comment on the computational cost of the present model is in order. As just seen, the computational cost of the present compressible model is one order higher than its incompressible counterpart, the *DdQ*$3^{d}$ lattice. However, for compressible (or high Mach number) simulations, the time step δtU/L, where *U* and *L* are characteristic velocity and length, is in general one to two orders of magnitude larger than for the incompressible flow simulations since the time step is directly proportional to the characteristic Mach number. This implies that no significant increase of computational cost may be observed in present simulations with respect to incompressible LBM.

It is also interesting to analyze the influence of computational load with the variation of the flow field (ρ, u and *T*) for the numerical evaluation of the equilibrium. In fact, since the Newton–Raphson solver for the equilibrium employs previous time step LM as an initial guess, one would expect that a high time variability of the flow could influence the performance. We propose here to measure the influence of the time variability of the flow field with the simulation of compressible decaying isotropic turbulence similar to the one presented in [17], for different initial turbulent Mach numbers ${\mathrm{Ma}}_{t}$. The initial turbulent Mach number is considered here as the quantitative measure of the time variability of the flow. In Table 3, we show the influence of the time-variability of the flow (in terms of ${\mathrm{Ma}}_{t}$) by measuring the computational time of the various decaying turbulence simulations. The lattice employed for the simulations is the *D*3*Q*$7^{3}$.

As one can notice, the computational time for the simulation increases only slightly with increasing turbulent Mach number. In fact, the computational time is increased at most by 7%, when the turbulent Mach number is quite high (the local Mach number may reach values above ${\mathrm{Ma}}_{\mathrm{loc}}≃2.0$).

#### 2.4.3. Numerical Equilibrium Accuracy

In this section, we analyze the numerical equilibrium accuracy in terms of deviation of the pertinent moments from their Maxwell–Boltzmann counterparts,

(32) $$\epsilon_{m}=\frac{M_{m}^{\mathrm{eq}}-M_{m}^{MB}}{M_{m}^{MB}}.$$

**Figure 2:**
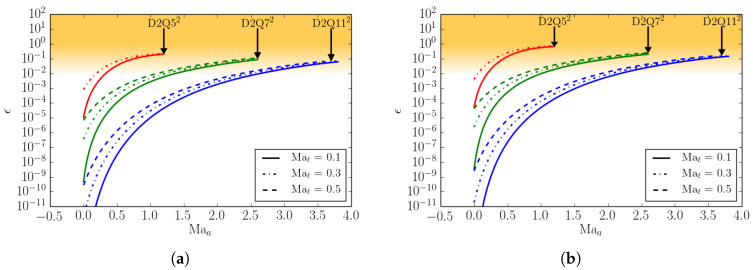
Deviation of the equilibrium moments from the Maxwell–Boltzmann values as a function of the turbulent and the advection Mach number ${\mathrm{Ma}}_{t}$ and ${\mathrm{Ma}}_{a}$, respectively, for three different lattices, *D*2*Q*$5^{2}$, *D*2*Q*$7^{2}$, and *D*2*Q*$11^{2}$. (**a**) The *x*-component of the third-order moment $q_{x}^{\mathrm{eq}}=\sum_{i=1}^{n}v_{ix}v_{i}^{2}f_{i}^{\mathrm{eq}}$; (**b**) The xx-component of fourth-order moment $R_{xx}^{\mathrm{eq}}=\sum_{i=1}^{n}v_{ix}v_{ix}v_{i}^{2}f_{i}^{\mathrm{eq}}$.

Superscript MB denotes the moments computed with the continuous Maxwell–Boltzmann distribution of a general moment of order *m*. In Figure 2, we compare the *D*2*Q*$5^{2}$ and *D*2*Q*$7^{2}$ lattices with respect to the errors in the equilibrium heat flux $q_{x}^{\mathrm{eq}}$ and in the equilibrium contracted fourth order moment $R_{xx}^{\mathrm{eq}}$ at various Mach numbers. Two types of Mach number are here defined: ${\mathrm{Ma}}_{t}$ stands for turbulent Mach number and ${\mathrm{Ma}}_{a}$ is the advection Mach number. The equilibrium is computed for a Mach number ${\mathrm{Ma}}_{a}$ in the *x*-direction plus a Mach number ${\mathrm{Ma}}_{t}$ superimposed in both the *x*- and *y*-directions. In the same plot, for completeness, the errors of the *D*2*Q*$11^{2}$ lattice are also shown.

It is important to note that the aforementioned moments need to be matched as accurately as possible by the given lattice in order to recover the correct thermo-hydrodynamic limit. One can notice that the errors in the moments remain small up to the advection Mach number ${\mathrm{Ma}}_{a}\leq 1.2$ for the *D*2*Q*$7^{2}$ lattice, while for the *D*2*Q*$5^{2}$ lattice the errors grow rapidly already in the subsonic regime. This implies that, in order to perform supersonic simulations, the minimum number of lattice velocities is $n=7^{D}$ in *D* dimensions. For this reason, from now on, we will consider the *D*2*Q*$7^{2}$ lattice as the minimal (among the lattices built by a direct product of the one-dimensional velocity sets) in order to perform compressible flow simulations.

**Figure 3:**
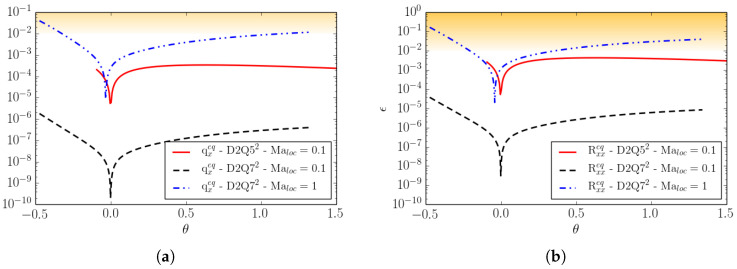
Deviation of the equilibrium moments from the Maxwell–Boltzmann values as a function of the reduced temperature θ. (**a**) The *x*-component of the third-order moment $q_{x}^{\mathrm{eq}}$; (**b**) The xx-component of the fourth-order moment $R_{xx}^{\mathrm{eq}}$. Results for the *D*2*Q*$5^{2}$ lattice at the local Mach ${\mathrm{Ma}}_{\mathrm{loc}}=0.1$ and for the *D*2*Q*$7^{2}$ lattice at local Mach ${\mathrm{Ma}}_{\mathrm{loc}}=0.1$ and ${\mathrm{Ma}}_{\mathrm{loc}}=1$ are shown.

In Figure 3, the same errors as for Figure 2 are plotted as a function of the reduced temperature θ, for two different lattices, *D*2*Q*$5^{2}$ and *D*2*Q*$7^{2}$, at different Mach numbers. Errors are shown within the reduced temperature range appropriate for the lattice as discussed in Section 2.3.

One can notice that, by increasing the number of velocities, the errors decrease by an order of magnitude, and the same is valid even when moving away from the reference temperature. Moreover, we can see that, by increasing the Mach number, the errors increase with the deviation from the reference temperature. However, considering that moderately strong shock waves have a reduced temperature jump of the order of Δθ≃0.3, we can state that supersonic flow simulations with shocks are permitted by the *DdQ*$7^{d}$ lattice.

#### 2.4.4. Equilibria Positivity

**Figure 4:**
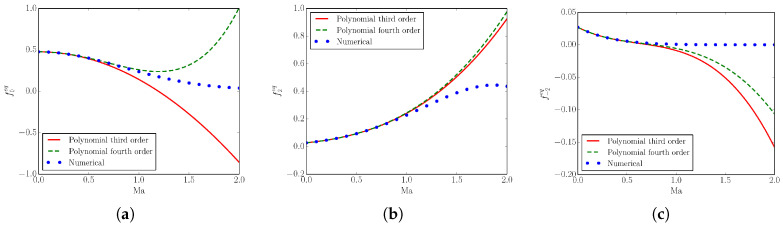
Equilibrium populations as a function of the Mach number. (**a**) $f_{0}^{\mathrm{eq}}$; (**b**) $f_{2}^{\mathrm{eq}}$; (**c**) $f_{-2}^{\mathrm{eq}}$. Line: Third-order polynomial approximation; Dash: Fourth-order polynomial approximation; Symbol: Accurate numerical evaluation.

In order to conclude this section, we point out a remarkable property of the numerical equilibrium. For the *DdQ*$7^{d}$ lattice discussed in Section 2.3, we provide a quantitative evidence of the difference between evaluating the equilibrium with the accurate numerical procedure and with a polynomial approximation. In Figure 4, we plot, for the one-dimensional case *D*1*Q*7, the equilibrium populations $f_{0}^{\mathrm{eq}}$, $f_{2}^{\mathrm{eq}}$ and $f_{-2}^{\mathrm{eq}}$ evaluated in three different ways as a function of the Mach number Ma: a third- and a fourth-order polynomial approximation (in terms of u) of equilibrium (30), and the accurate equilibrium (Newton–Raphson evaluation).

It is clear that, as the Mach number is increased, especially after the Mach number reaches the sonic value, the polynomial approximations start to diverge from the exact solution to the minimization problem, leading to accumulation of errors. Moreover, an increase of the order of approximation from third to fourth has only a minor influence on the convergence to the correct solution. Moreover, after a certain value of the Mach number, the populations evaluated with a polynomial approximation become negative, violating the positivity constraints imposed by the construction of the equilibrium. From Figure 4, it is possible to conclude that numerical evaluation of the equilibrium is not just more accurate, but it is also unavoidable for compressible lattice Boltzmann simulations.

However, the constraint of positivity on the entropic equilibrium comes along with a cost: as seen in Section 2.4.3, the equilibrium moments starts to deviate from their continuous Maxwell–Boltzmann counterparts when the Mach number is increased above a certain limit or/and the temperature deviates from the reference value $T_{0}$. In general, from the above analysis of errors as a function of Mach number Ma and reduced temperature θ, we observe that the *DdQ*$7^{d}$ lattice has all the minimal characteristics required to run compressible flows simulations, i.e., accuracy at relatively high Mach numbers and a wide enough temperature range to guarantee positivity of the populations. In Section 3, we extend the operation window in terms of Mach number and temperature range by simply modifying the lattice configuration and without increasing the number of the discrete velocities.

## 3. Extension of Operation Domain

As outlined in the previous section, the accuracy for a given lattice is restricted in terms of deviation of a local Mach number and of the temperature from their reference values (reference Mach Ma=0, and reference temperature $T_{0}$). In this section, we address this issue by retaining the same number of discrete velocities. A brute force way to overcome these issues would be, in fact, to increase the number of discrete velocities. For example, when going from the *DdQ*$7^{d}$ lattice to the *DdQ*$11^{d}$ lattice, the deviation from Maxwell–Boltzmann moments reduces; between an advection Mach number of ${\mathrm{Ma}}_{a}=1$ and ${\mathrm{Ma}}_{a}=2$, the reduction of errors is one to two orders of magnitude, see Figure 2. However, as also demonstrated in Table 2, the computational cost increases at least linearly with the number of populations, so that the simulation with the *D*2*Q*$11^{2}$ and especially the *D*3*Q*$11^{3}$ would quickly become prohibitively demanding. In the next two sections, we suggest how the Mach number can be increased and how the temperature range can be extended without enlarging the number of velocities. To that end, the lattice *DdQ*$7^{d}$ is considered, as it has already been benchmarked for compressible flows simulations [17,18].

### 3.1. Shifted Lattices

We follow [19] in order to extend the Mach number range. This method is particularly useful when the mean flow has a preferential direction, for example, in the simulation of supersonic flow around obstacles, or a flow with stationary shocks and mean cross-flow like the simulation of shock–vortex or shock–turbulence interaction.

We wish to challenge the notion of a reference frame at rest; instead, we prefer to define the equilibrium in a frame moving, say, with the velocity *U* in the *x*-direction. This is achieved by considering a *shifted* discrete velocity set $V_{n}^{\prime }$ with the velocities

(33) $$v_{i}^{\prime }=v_{i}+U,\;v_{i}\in V_{n},$$

and finding the shifted weights $w_{i}\left(U,T\right)$ by matching the moments of the Maxwellian computed at velocity *U* and temperature *T*, $f_{v}^{M}\left(U,T\right)$:

(34) $$f_{v}^{M}\left(U,T\right)=\sqrt{\frac{1}{2\pi T}}\exp\left\{-\frac{{\left(v-U\right)}^{2}}{2T}\right\}.$$

Key observation of [19] is that the weights $w_{i}\left(U,T\right)$ are Galilean invariant: For any set $V_{n}$ and for any shift *U*, we have

(35) $$w_{i}\left(U,T\right)=w_{i}\left(0,T\right).$$

The proof of (35) follows immediately from Galilean invariance of the moments of the Maxwellian; see [19].

**Figure 5:**
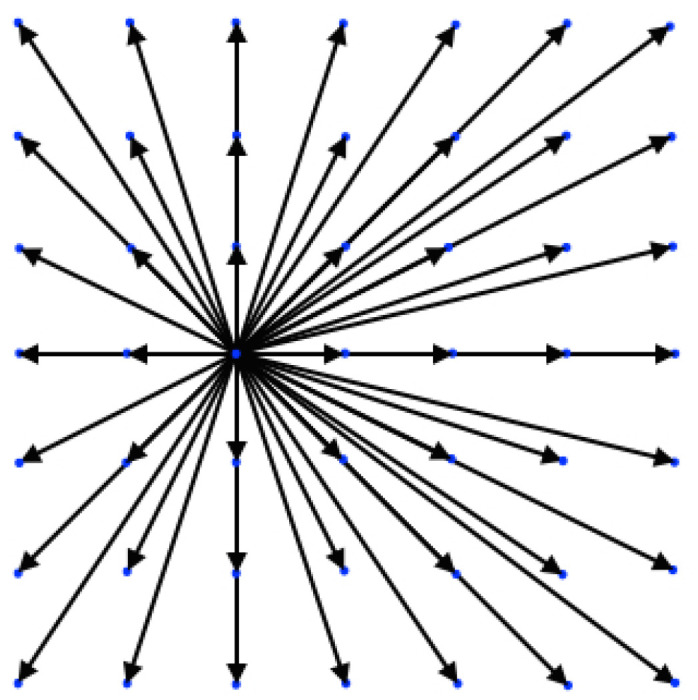
Visualization of the shifted *D*2*Q*49 lattice with U=1.

Galilean invariance of the weights (35) has a number of important implications. First, the construction of the discrete velocities through tensor products in higher dimensions remains as before. For example, in two dimensions, the shift *U* in the *x*-direction corresponds to tensor product $V_{nx}^{\prime }⊗V_{ny}$. We shall use this example below. Second, the symmetric integer velocity sets generate space-filling lattices. This is crucial for the realization of the time-marching LB scheme (1). Now, if the shift *U* is itself integer, the corresponding shifted lattice is also space-filling. For example, with U=1, the shifted set $V_{7}^{\prime }$ becomes $V_{7}^{\prime }=\left\{-2,-1,0,1,2,3,4\right\}$. The shifted lattice, with the discrete velocities $V_{7x}^{\prime }⊗V_{7y}$ is shown in Figure 5. Next, the weights corresponding to the velocities of the shifted lattice are the same as for the symmetric case: $W_{i}\left(U,T\right)=w_{ix}\left(0,T\right)w_{iy}\left(0,T\right)$. Moreover, since the weights do not change under a transformation to a co-moving reference frame, the entropy function is Galilean invariant, and is given by Equation (9). Consequently, the equilibrium populations are form-invariant. They are found by the same minimization techniques as in the familiar symmetric case.

**Figure 6:**
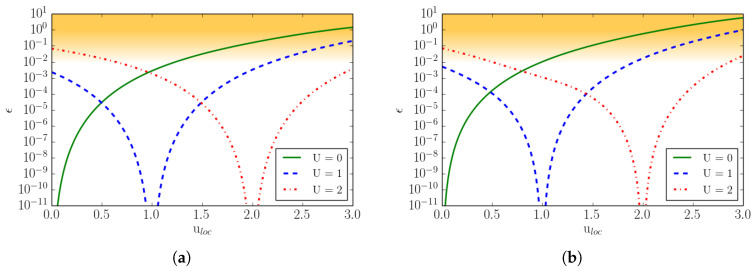
Deviation of the equilibrium moments from the Maxwell–Boltzmann values as a function of the *x*-component of the local velocity. (**a**) The third-order moment $q_{x}^{\mathrm{eq}}$; (**b**) The fourth-order moment $R_{xx}^{\mathrm{eq}}$. Line: Non-shifted lattice, U=0; Dash: Shifted lattice, U=1; Dotted-dash: Shifted lattice, U=2.

The errors associated with the shifted lattices are shown in Figure 6, for the same quantities as presented in Figure 2. Errors for the lattice with no shift, and with a shift U=1 and U=2 are shown. The errors, in Figure 2, are plotted now as a function of the $u_{x}$ velocity in order to show the effect of the lattice shift. When the velocity matches the shift velocity, the errors in the moments vanish.

**Figure 7:**
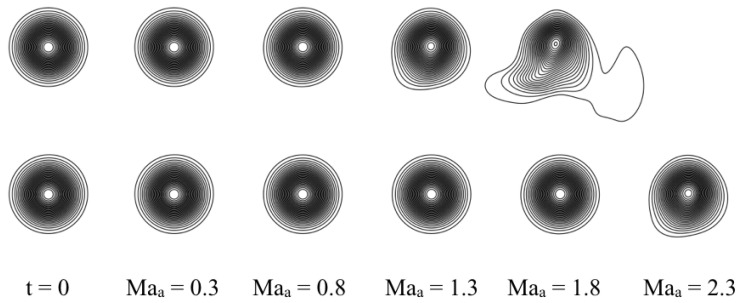
Vortex advection comparison at different advection Mach number. Top row: symmetric lattice. Bottom row: shifted lattice with U=1.

In order to understand the effect of the shifted lattice, we run test simulations of a vortex advected at different Mach numbers. After a certain Mach number, the non-shifted lattice is expected to feature some numerical errors due to the lack of Galilean invariance as noted in Figure 6. Due to these numerical errors, the vortex is no longer advected correctly and starts to deform. The same is expected also for the shifted lattice, but around a higher Mach number. This is demonstrated in Figure 7 for a characteristic vortex Mach number of ${\mathrm{Ma}}_{v}=0.5$ and different advection Mach numbers, ${\mathrm{Ma}}_{a}=\left\{0.3,0.8,1.3,1.8,2.3\right\}$, by means of pressure contours.

**Figure 8:**
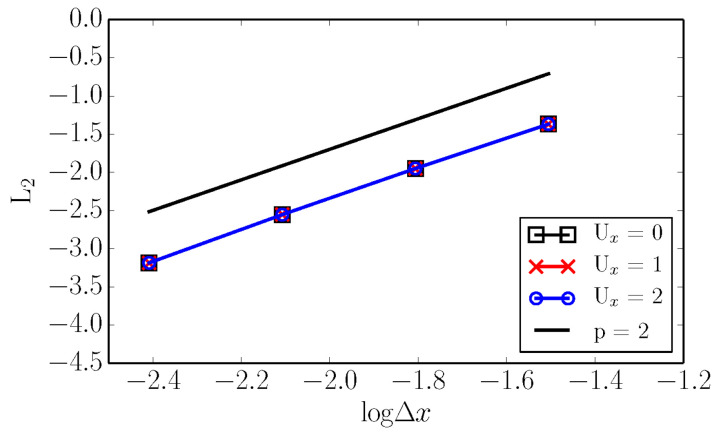
Grid convergence study for the Green–Taylor vortex. Results are shown for non-shifted lattice and lattices with the shift U=1 and U=2.

Second order convergence was checked for lattices without and with the shifts U=1 and U=2, by simulating the Green–Taylor vortex with a mean advection speed of U=0, U=1, U=2, respectively. We evaluate the $L_{2}$ norm of the error defined as,

(36) $$L_{2}=\sqrt{\frac{\sum_{i}{\left(u_{i,LBM}-u_{i,ref}\right)}^{2}}{\sum_{i}u_{i,ref}^{2}}},$$

where $u_{i,LBM}$ is the velocity at sample *i* with the present simulations, and $u_{i,ref}$ is the velocity of computed from analytical solution. The result of the grid convergence study is presented in Figure 8 and shows no difference between the results of a non- and shifted lattices.

### 3.2. Lattices with Increased Temperature Range

The range of temperatures supported by the lattice is increased based on the following observation: By considering a generic velocity set $V_{7}=\left\{0,\pm 1,\pm 2,\pm r\right\}$, where the above lattice for compressible flow is obtained for r=3 [17], we notice that, by increasing the longest lattice link to r=4, the temperature range increases significantly while the lattice still belongs to the same hierarchy of the admissible higher-order lattices. In Table 4, we report the minimum $T_{min}$ and maximum $T_{max}$ temperatures, the reference temperature $T_{0}$, the reduced temperatures $\theta_{min}$ and $\theta_{max}$, and the temperature ratio $T_{max}/T_{min}$.

**Figure 9:**
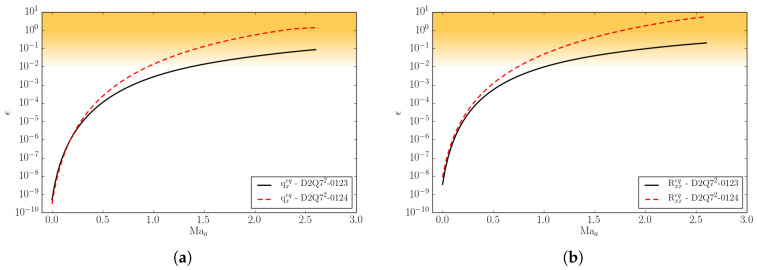
Deviation of the equilibrium moments from the Maxwell–Boltzmann values as a function of Mach number. (**a**) The third-order moment $q_{x}^{\mathrm{eq}}$; (**b**) The fourth-order moment $R_{xx}^{\mathrm{eq}}$. Line: The *D*2*Q*$7^{2}$-0123 lattice. Dash: The *D*2*Q*$7^{2}$-0124 lattice.

The temperature ratio $T_{max}/T_{min}$ is the most important parameter to characterize the temperature range. An increase of the temperature range, however, comes at the expense of increased errors in the higher-order moments $q_{\alpha }^{\mathrm{eq}}$ and $R_{\alpha \beta }^{\mathrm{eq}}$ when the Mach number is increased, as shown in Figure 9.

**Figure 10:**
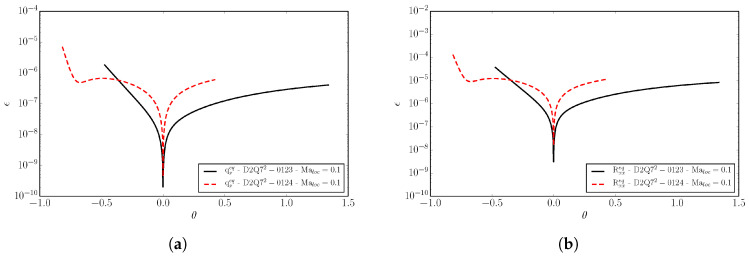
Deviation of the equilibrium moments from the Maxwell–Boltzmann values as a function of the reduced temperature θ, at the local Mach number ${\mathrm{Ma}}_{\mathrm{loc}}=0.1$. (**a**) The third-order moment $q_{x}^{\mathrm{eq}}$. (**b**) The fourth-order moment $R_{xx}^{\mathrm{eq}}$. Line: The *D*2*Q*$5^{2}$-0123 lattice; Dash: The *D*2*Q*$7^{2}$-0124 lattice.

In Figure 10, the same errors are shown as a function of the temperature. At the specified Mach number ${\mathrm{Ma}}_{\mathrm{loc}}=0.1$, the errors remain small over the entire temperature range for both lattices.

Summarizing, since the errors of the *D*2*Q*$7^{2}$-0124 lattice are higher than for the *D*2*Q*$7^{2}$-0123 lattice, the latter should be the preferred choice. However, it may happen that for certain simulations the temperature range that needs to be achieved is greater than the maximum allowed by the *DdQ*$7^{d}$-0123 lattice, while the flow remains subsonic. In such cases, the *DdQ*$7^{d}$-0124 lattice may become particularly useful. We demonstrate shall demonstrate this with the simulation of Richtmyer–Meshkov instability in the next section.

## 4. Numerical Results

In this section, we validate the model, the shifted lattices, and the lattice with greater temperature range by means of five different set-ups: the supersonic inviscid flow around a diamond airfoil, the flow around the Busemann biplane from subsonic to supersonic Mach numbers, supersonic viscous flow around a NACA0012 airfoil, and the interaction of a vortex and of a pair of vortices with a shock wave. The increased temperature range lattice is validated by means of the Richtmyer–Meshkov simulation. Finally, we demonstrate a three-dimensional simulation of a transonic flow over the Onera M6 wing. The boundary conditions developed in [18] are employed for all the validations.

### 4.1. Supersonic Diamond Airfoil

**Figure 11:**
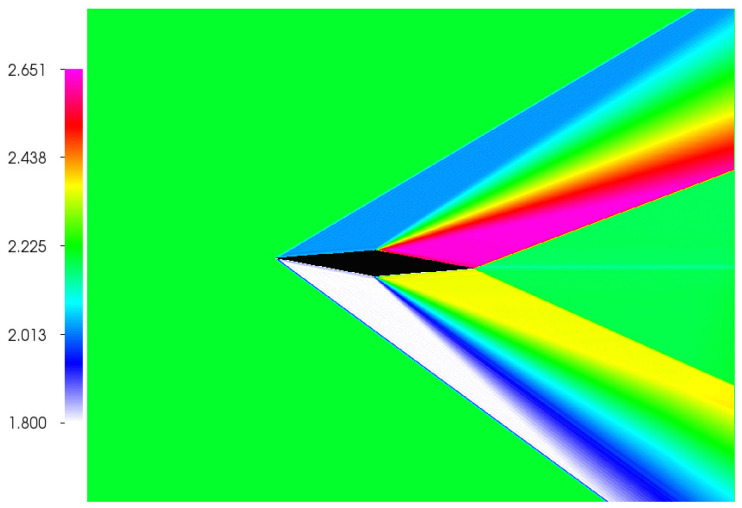
Steady state solution of the Mach number distribution around a diamond-shaped airfoil at the inlet Ma=2.2, Re=106 and $\mathrm{AoA}=3^{\circ }$.

We start by showing, in Figure 11, the two-dimensional simulation of the supersonic flow field around a diamond-shaped airfoil. The inflow Mach number is set to ${\mathrm{Ma}}_{\mathrm{in}}=2.2$, while the Reynolds number is Re=106 based on the chord length, and the chord is resolved with C=500 grid points; the wedge angle is $\subseteq =12^{\circ }$. This setup has been already discussed in detail in [18] for the non-shifted (standard) lattice where the maximum Mach number of the order ${\mathrm{Ma}}_{\mathrm{in}}=1.5$ could be achieved. Here, using the shifted lattice with U=1 and the same boundary, inlet and outlet conditions, we demonstrate that the simulation can be performed at higher Mach number without any change in the algorithm.

One can notice the characteristic oblique shocks appearing at the leading and trailing edges of the airfoil and the expansion wave propagating from the midspan of the airfoil. In Table 5, we compare the results obtained with the ELBM solver for pressure distribution, Mach number distributions, and oblique shock angles with analytical solution using thin airfoil approximation [29].

Agreement between theory and simulation is excellent and demonstrates the accuracy of the shifted lattice.

### 4.2. Busemann Biplane

**Figure 12:**
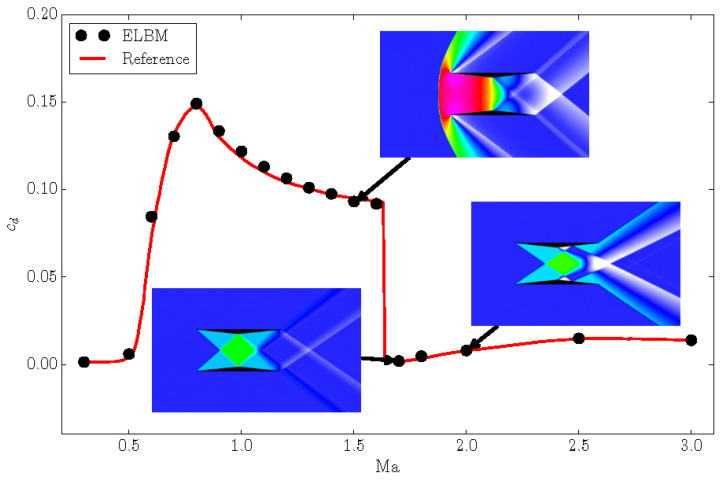
Drag coefficient $c_{d}$ as a function of the free stream Mach number for the Busemann biplane simulations. Reference: [30]. Inset: snapshots of the pressure distribution around the biplane for three different Mach numbers: Ma=1.5, top; Ma=1.7, bottom left; Ma=2.0, bottom right. The pressure is shown between $p_{min}=0.5$ (white) and $p_{max}=1.9$ (pink), in lattice units.

In order to validate the model and the shifted lattices over a wide range of Mach numbers, we show here the simulation of the Busemann biplane [30] in its two-dimensional configuration. The Busemann biplane is composed of two half-diamond airfoils as shown in the insets in Figure 12, and was originally conceived in order to reduce the wave-drag for supersonic flight. It is well known that conventional airfoils experience a dramatic increase of the drag coefficient $c_{d}$ near sonic conditions and, after that, with the Mach number increasing, the drag coefficient starts to progressively decrease again.

The same happens in the case of the Busemann biplane where the drag coefficient increases dramatically near sonic condition and, similar to a conventional diamond airfoil, it starts to decrease when the Mach number is further increased. However, for the Busemann biplane, after a critical Mach number, the drag coefficient drops well below that of the normal diamond-shaped airfoil. This is shown in Figure 12 where we compare the drag coefficient computed with the ELBM model to the simulation reported in [30]. We performed simulations of the Busemann biplane in its simplest configuration, with two symmetric half-diamond airfoils with zero angle of attack. The chord of the airfoil was resolved with C=400 grid points. The range of simulated Mach numbers varies tenfold from ${\mathrm{Ma}}_{\min}=0.3$ to ${\mathrm{Ma}}_{\max}=3.0$. Along with the increasing Mach number, different lattices were employed: from Mach number ${\mathrm{Ma}}_{\min}=0.3$ to Ma=1.3, the conventional symmetric lattice *D*2*Q*49 (U=0) was used. From Ma=1.4 to Ma=2.0, the lattice with a shift of U=1 was considered. Finally, for the two highest Mach numbers, a lattice with a shift of U=2 in the *x*-direction was chosen. The results of the ELBM model agree well with the simulations of [30]. In particular, the sensitive drag crises happening between Ma=1.6 and Ma=1.7 are well captured. This condition of drag crises corresponds to a free-stream Mach number at which the oblique shock waves developing from the leading edges reattach to the mid-point of the opposite airfoil, thus reflecting nearly onto its own trailing edge.

The inset in Figure 12 shows the pressure distribution around the biplane at three representative Mach numbers: before, near, and after the drag crises. At Ma=1.5, before the drag crises, a detached bow shock is formed in front of the airfoils, responsible for a dramatic rise of the pressure which in turn increases the wave drag of the biplane. After the Mach number becomes sufficiently high, the bow shock approaches gradually the leading edge of the airfoils and finally it transforms into two attached oblique shocks at leading edges. At this point, the drag dramatically decreases. The minimum drag coefficient is obtained at about Ma=1.7 which is represented by the lower left inset. As anticipated above, for this case, the oblique shocks of the leading edges attain an optimal configuration in terms of drag coefficient. This is due to a combination of the geometric angle of the oblique shock wave and the geometry of the airfoil; at this Mach number, the oblique shock matches the middle edge of the opposite half-diamond airfoil. This has two effects: an influence on the expansion wave formed there, and a reflection of the oblique shock which reaches exactly the trailing edge of the proper airfoil [30]. This perfect matching induces the minimum drag coefficient reported in Figure 12. When the Mach number is further increased, e.g., like in the case represented by the lower right inset, Ma=2.0, the oblique shock configuration does not match the geometry of the biplane any longer, and the drag coefficient starts to increase again. The present example clearly demonstrates the capability of the model to accurately perform simulations over a wide range of Mach numbers. This highlights the typical use of the shift velocity: as the mean Mach number increases, we increase the shift velocity, thus minimizing the errors arising from the non-optimal use of the standard reference frame.

### 4.3. Supersonic NACA0012 Airfoil

**Figure 13:**
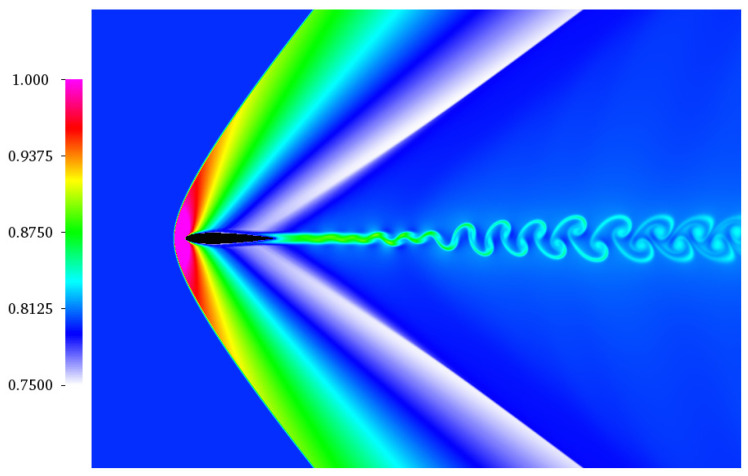
Snapshot of the temperature around the NACA0012 airfoil with a free stream Mach of Ma=1.5, Reynolds number Re=104, and angle of attack $\mathrm{AoA}=0^{\circ }$.

A further validation of the model for high Mach flow is carried out by the simulation of viscous supersonic flow field around a NACA0012 airfoil. Here, we set the free stream Mach number ${\mathrm{Ma}}_{\infty }=1.5$, the Reynolds number Re=104, and zero angle of attack $\mathrm{AoA}=0^{\circ }$. The chord was resolved with a uniform grid of C=800 points. Figure 13 shows the temperature distribution around the airfoil at a time $t/(C/U_{in})=232.3$.

**Figure 14:**
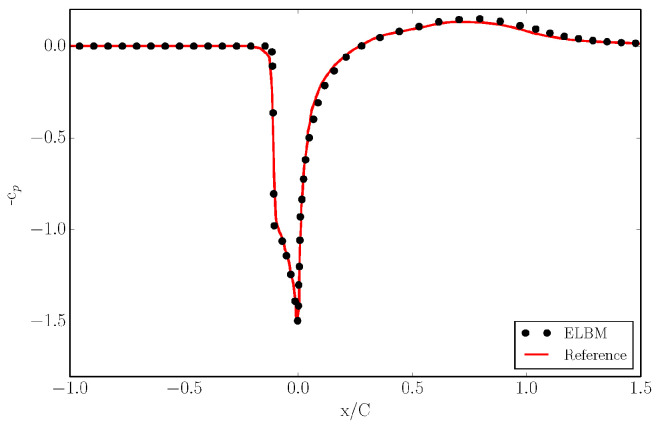
Pressure coefficient in front of the airfoil, on the airfoil surface and behind the airfoil for the simulation of the NACA0012 airfoil at free stream Mach of Ma=1.5, a Reynolds number Re=104, and an angle of attack $\mathrm{AoA}=0^{\circ }$.

One can observe the typical bow shock forming in front of the airfoil for supersonic conditions as well as the the oblique shocks starting as a lambda-shock from the trailing edge. Moreover, due to the shear layer developing from the trailing edge of the airfoil, vortex shedding is initiated downstream. In order to validate the simulation, we plot in Figure 14 the pressure coefficient along the upstream, airfoil surface and downstream direction and we compare it with reference [31].

The plot demonstrates the capability of ELBM on shifted lattices to correctly compute the pressure distribution. It is clear that the introduction of stationary walls is possible also for shifted lattices which assume a mean flow. This is due to the presence of a rest particle that resides at the grid node.

### 4.4. Shock–Vortex Interaction

In the following, we present simulation of a well-studied problem of vortex–shock interaction where the *D*2*Q*$7^{2}$ lattice with a shift U=1 was used. We compare our solutions to the DNS simulation of [32]. A two-dimensional vortex characterized by a vortex Mach number ${\mathrm{Ma}}_{v}$ is advected at a Mach number ${\mathrm{Ma}}_{a}$ and subsequently interacts with a stationary shock wave. The advection Mach number corresponds to the relative Mach number of the shock with respect to the upstream velocity, ${\mathrm{Ma}}_{s}={\mathrm{Ma}}_{a}$; the shock is kept stationary. The initial flow field of the vortex is given by:

(37) $$u_{\theta }\left(r\right)=\sqrt{\gamma T}Ma_{v}r\exp\left(\left(1-r^{2}\right)/2\right),\;u_{r}=0.$$

Here, $u_{\theta }$ and $u_{r}$ are the tangential and the radial velocity of the vortex, respectively, and *r* is the reduced radius $r=r^{{}^{\prime }}/R$, with *R* the characteristic radius. Pressure and density distributions are initialized as follows:

(38) $$\begin{array}{cc} p(r) & =\frac{1}{\gamma }{\left[1-\frac{\gamma -1}{2}Ma_{v}^{2}\exp\left(1-r^{2}\right)\right]}^{\gamma /(\gamma -1)}, \end{array}$$

(39) $$\begin{array}{cc} \rho (r) & ={\left[1-\frac{\gamma -1}{2}Ma_{v}^{2}\exp\left(1-r^{2}\right)\right]}^{1/(\gamma -1)}. \end{array}$$

The vortex flow field is superimposed on a constant flow with ${\mathrm{Ma}}_{a}$ upstream of the shock front; see [32] for the details of the numerical set-up. Three different configurations were simulated: ${\mathrm{Ma}}_{a}=1.2$, ${\mathrm{Ma}}_{v}=0.5$ and Re=400; ${\mathrm{Ma}}_{a}=1.2$, ${\mathrm{Ma}}_{v}=0.25$ and Re=800; ${\mathrm{Ma}}_{a}=1.05$, ${\mathrm{Ma}}_{v}=0.25$ and Re=400. The Reynolds number was defined as Re=a∞R/∘, with the speed of sound $a_{\infty }=\sqrt{\gamma T}$.

**Figure 15:**
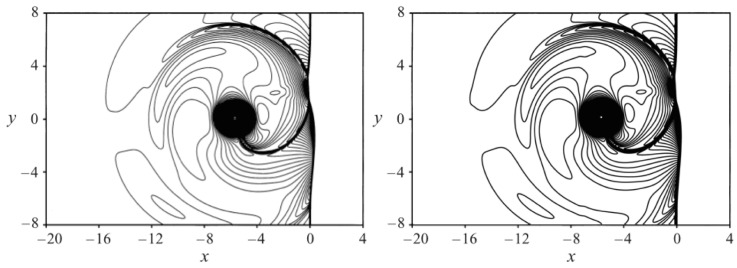
Snapshot of the density in the vortex–shock interaction simulation. Left: DNS [32]; Right: ELBM. ${\mathrm{Ma}}_{a}=1.2$, ${\mathrm{Ma}}_{v}=0.5$ and Re=400. Contour levels are from $\rho_{min}=0.92$ to $\rho_{max}=1.55$ with an increment of Δρ=0.0053.

In Figure 15, the contours of density ρ for the case ${\mathrm{Ma}}_{a}=1.2$, ${\mathrm{Ma}}_{v}=0.5$ and Re=400 are compared to the results reported in [32] at the time instance $t^{\prime }=t/\left(R/a_{\infty }\right)=8$. One can notice the deformed shock at x=0, the deformed vortex at x≃−5 and y≃0, and the reflected shocks developing from the original planar shock, one of which is still connected to the vortex. The density contours show in general a good comparison between DNS and ELBM simulations.

**Figure 16:**
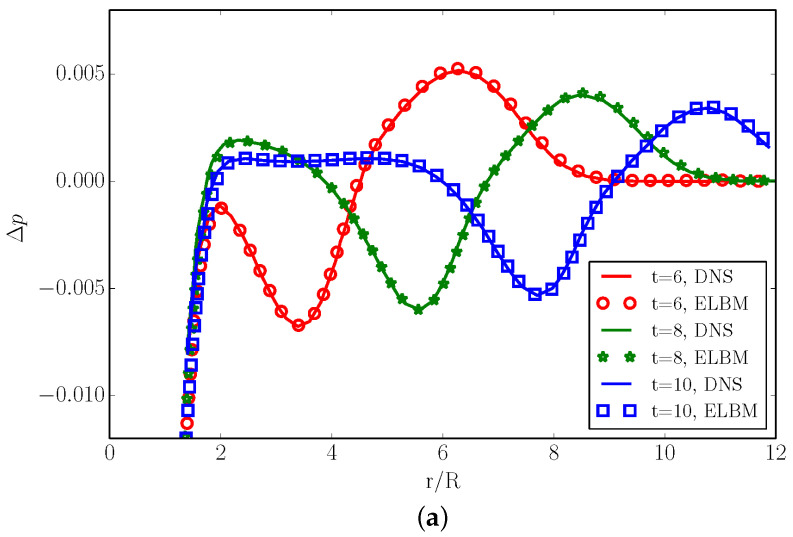

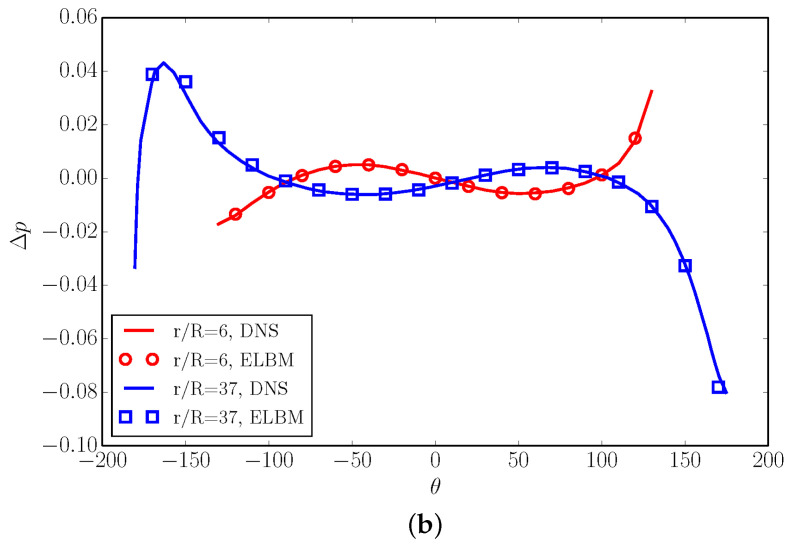
The sound pressure distribution Δp in the radial and tangential directions for the case ${\mathrm{Ma}}_{a}=1.2$, ${\mathrm{Ma}}_{v}=0.25$ and Re=800. (**a**) The radial sound pressure distribution Δp measured at an angle $\theta =-45^{\circ }$ with respect to the *x*-axis. Times: $t^{\prime }=6$, $t^{\prime }=8$, $t^{\prime }=10$. (**b**) The tangential sound pressure distribution Δp measured at two radii, r=6 and r=3.7. Time: t=6. Symbol: ELBM. Line: DNS [32].

In Figure 16, we compare the radial and tangential sound pressure distribution, respectively. The sound pressure is defined as $\Delta p=\left(p-p_{s}\right)/p_{s}$, where $p_{s}$ is the pressure behind the shock wave. The radial sound pressure distribution was measured in cylindrical coordinates with the origin at the vortex center, at an angle $\theta =-45^{\circ }$ with respect to the *x*-axis and at different times $t^{\prime }=6$, $t^{\prime }=8$, $t^{\prime }=10$. The tangential sound pressure distributions were measured in the tangential coordinate at two different radii r=6 and r=37, at time t=6. From the radial distribution, one can notice how both the sound precursor (upper sound pressure peak) and the second sound propagate radially from the vortex center with time. Moreover, the peak sound $\Delta p_{m}$ corresponding to the maximum peak pressure decays with time, for both the precursor and second sound. From the tangential distribution, it is possible to observe the quadrupole nature of the sound generated by the vortex–shock interaction [32]: the second sound measured at r=3.7 has the opposite sign with respect to the precursor at r=6.

**Figure 17:**
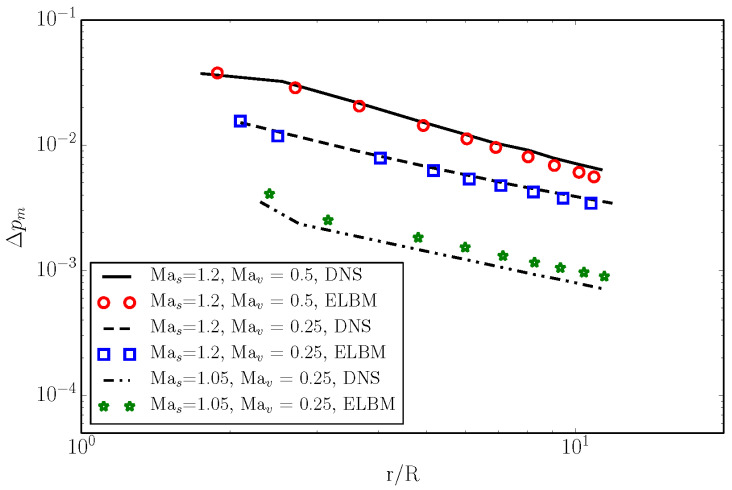
The decay of the peak sound pressure at different Mach numbers. Symbol: ELBM. Lines: DNS [32].

In Figure 17, we plot the peak sound pressure $\Delta p_{m}$ of the precursor measured at $\theta =-45^{\circ }$ at different times, and the corresponding radial position. We can remark that the amplitude of the generated sound grows with the increase of both shock and vortex Mach numbers.

**Figure 18:**
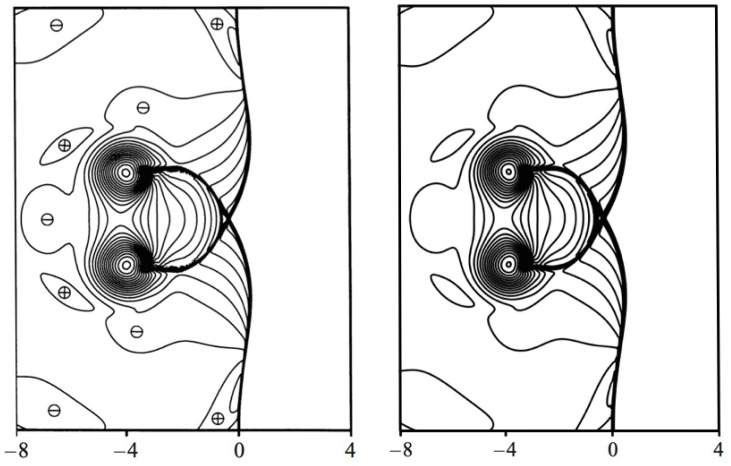
Snapshot of the sound pressure, ${\mathrm{Ma}}_{a}=1.2$, ${\mathrm{Ma}}_{v}=0.5$ and Re=400. Left: DNS [32], ⊕ and ⊖ indicate positive and negative sound pressure, respectively; Right: ELBM. Contour levels are from $\Delta p_{min}=-0.459$ to $\Delta p_{max}=0.301$ with an increment of Δρ=0.027.

Finally, we show in Figure 18 the simulation of a pair of vortices interacting with a shock wave using the same parameters as in Figure 15: ${\mathrm{Ma}}_{a}=1.2$, ${\mathrm{Ma}}_{v}=0.5$, and Re=400. We compared the sound pressure of the ELBM simulation with the DNS of [32]. In addition, in this case, the comparison shows good agreement between the DNS and the ELBM method. This simulation indicates a low artificial dissipation of ELBM.

Before proceeding with the next set-up, we would like to highlight the following observation about the non-dimensional time step of our simulations as compared to DNS: The non-dimensional time step for the present ELBM is $\delta t\cdot a_{\infty }/R=1.67\times 10^{-2}$, while, for the reference DNS [32], it is $\Delta t\cdot a_{\infty }/R=1.75\times 10^{-4}$. This shows one of the advantages of ELBM, requiring two orders of magnitude less time steps as compared to the DNS. Strong coupling of lattice Boltzmann time step with the advection velocity (or Mach number) is a disadvantage for the LBM for low Mach number applications; however, this coupling becomes an advantage for compressible flows.

### 4.5. Richtmyer–Meshkov Instability

We proceed to the validation of the lattice with increased temperature range and consider the simulation of Richtmyer–Meshkov instability (RMI) [33]. In order to reproduce the experiment of [34], a minimum temperature ratio of $T_{max}/T_{min}≃4.5$ has to be allowed by the lattice; therefore, the *D*2*Q*$7^{2}$-0123 lattice cannot be used and the *D*2*Q*$7^{2}$-0124 lattice of Section 3.2 has to be employed instead.

In the RMI problem, a shock wave characterized by a shock Mach number ${\mathrm{Ma}}_{s}$ is passing through a stratified fluid with a density interface of a sinusoidal shape with an initial amplitude $a_{0}$ and characterized by the pre-shock Atwood number $A=\left(\rho_{2}-\rho_{1}\right)/\left(\rho_{1}+\rho_{2}\right)$. Here, $\rho_{1}$ and $\rho_{2}$ are the density upstream and downstream of the interface. After the shock front passes through the interface, the amplitude starts growing and the RMI develops. Experimental conditions of [34] were adopted in the simulation: the incident shock strength was ${\mathrm{Ma}}_{s}=1.21$ while the pre-shock Atwood number was A=0.601. The fluid was characterized by a mean adiabatic exponent γ=1.276. The initial interface amplitude is $ka_{0}=0.21$, where *k* is the wavenumber of the perturbation k=2π/λ. For further details, the reader is referred to [34]. In the simulation, we adopted λ=1878 grid points in the transversal direction.

**Figure 19:**
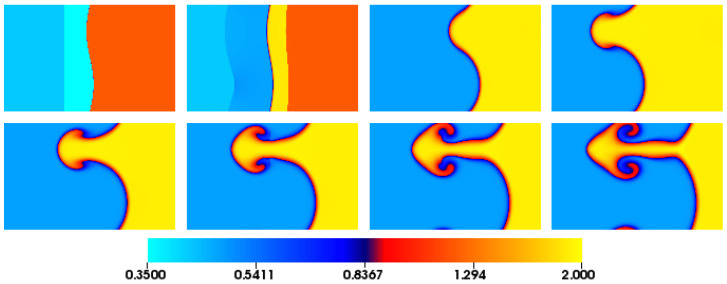
Snapshots of the evolution of the density for the Richtmyer–Meshkov instability. Time instants from left to right and from top to bottom; Top row: τ=−0.03, τ=0.09, τ=1.13, τ=2.29. Bottom row: τ=3.44, τ=4.60, τ=5.76, τ=6.92.

In Figure 19, we report eight snapshots of the evolution of the density. The normalized time τ is defined as $\tau =\left(t-t_{0}\right)ku_{0}$, where $u_{0}$ is the flow speed behind the density interface after the shock wave has passed through it. The initial conditions are represented by τ=−0.03 at which the shock wave is on the left of the sinusoidal density interface. After the passage of the shock, as shown for τ=0.09, a part of the shock travels in the same direction while the other part is refracted back upstream (see left interface). During the passage of the shock, vorticity is deposed near the density interface, thus initiating the growth of the amplitude of the sinusoidal interface, τ = 1.13–2.29. After the initial growth, the instability presents the typical formation of a mushroom-like plume, τ = 3.44–6.92.

**Figure 20:**
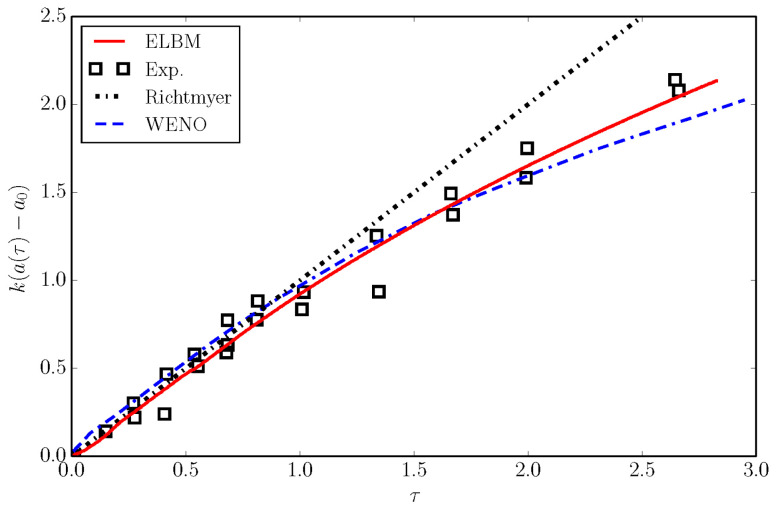
Normalized amplitude growth rate for the Richtmyer–Meshkov instability simulation as a function of normalized time. Solid line: ELBM; Symbol: the experiment [34]; Dashed line: the WENO simulation [35]; Dotted line: the original analytical prediction of Richtmyer [36] for the initial growth.

The simulation was validated by measuring the non-dimensional growth rate of the instability a(τ) and was compared with the experimental measurements of [34] and with the WENO simulations of [35]. WENO results were made non-dimensional with the experimental parameters ($u_{0}=628$ cm/s). Results are reported in Figure 20 and show a good match of the ELBM simulation with the experiment.

### 4.6. Transonic Onera M6 Wing

Our final example is the simulation of the transonic, inviscid flow field around the Onera M6 wing being reported. This kind of swept wing of intermediate aspect ratio is the standard benchmark for numerical methods for transonic three-dimensional flows, also thanks to the formation of a significant vortex above the wing tip which interacts with the lambda-shock forming due to the transonic conditions. The inflow Mach number was set to Ma=0.839 while the Reynolds number was Re=11.7×106 and angle of attack $\mathrm{AoA}=3.06^{\circ }$. The mean chord was resolved with $C_{m}=108.9$ grid points. Details of wing geometry and the set-up can be found in [37]. ELBM simulation was performed with the *D*3*Q*$7^{3}$ lattice.

**Figure 21:**
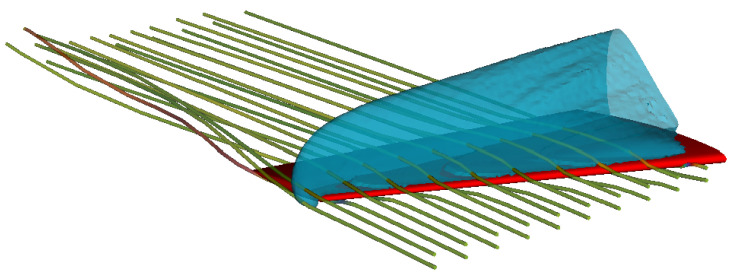
Snapshot of the flow field around the Onera M6 wing in a transonic flow at Ma=0.839, Re=11.7×106 and $\mathrm{AoA}=3.06^{\circ }$—the streamlines colored by vorticity and the iso-surface of the sonic condition are shown.

In Figure 21, an overview of the flow field is shown by streamlines colored by vorticity, and by the iso-surface of sonic condition. The flow field demonstrates the features typical of the transonic flow around a three-dimensional finite wing: at the tip of the wing, a characteristic tip vortex is formed, demonstrated by the helical pattern of the streamlines and with the increase of the vorticity (from green to red). Moreover, a lambda-shock forms on the upper part of the wing, shown by the sonic iso-surface which divides the domain into supersonic and subsonic regions. The shock, furthermore, continues toward the tip of the wing and reaches also the lower part; this is consistent with the results reported in the literature [37,38].

**Figure 22:**
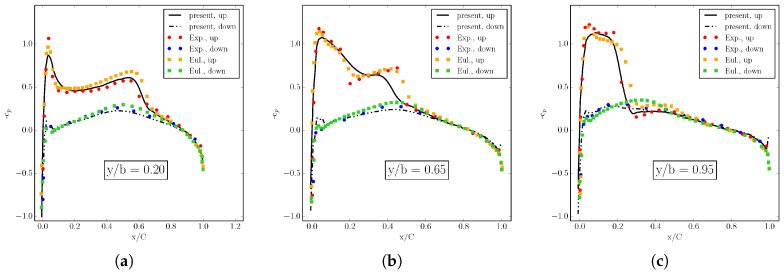
Pressure coefficient $c_{p}$ on the Onera M6 wing at three wing sections in the stream-wise direction. (**a**) y/b=0.2; (**b**) y/b=0.65; (**c**) y/b=0.95. Line: ELBM. Symbol: Experiment and Euler solver [37].

**Figure 23:**
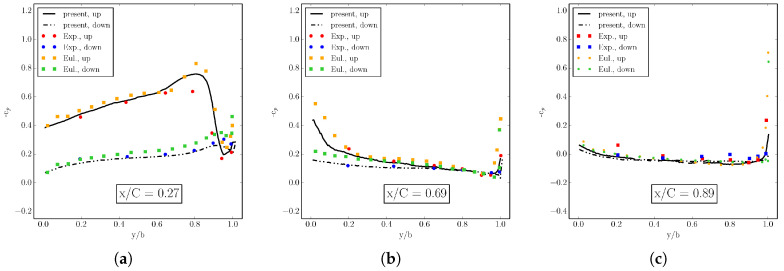
Pressure coefficient $c_{p}$ on the Onera M6 wing at three sectional span-wise positions. (**a**) x/C=0.27; (**b**) x/C=0.69; (**c**) x/C=0.89. Line: ELBM. Symbol: Experiment and Euler solver [37].

The shock formation is also revealed by the sectional stream-wise plots of pressure coefficient $c_{p}$ shown in Figure 22, where ELBM results are compared to the experiment and Euler solver simulations [37]. In particular, the pressure coefficient profiles along the span-wise direction reveal the formation of the lambda-shock on the upper wing surface: the upstream leg of the lambda-shock, located near the leading edge, is the foot of a three-dimensional shock, whereas the downstream leg of the lambda-shock is a 3D shock terminating the local supersonic region (see $c_{p}$ distributions at y/b=0.2 and y/b=0.65). Near the tip of the wing, the two shocks merge into a single one due to the presence of a strong tip vortex influencing the flow on the upper side of the wing (see the $c_{p}$ distribution at y/b=0.95). Even more insight into the characteristic of the flow field is provided by the sectional span-wise plots of $c_{p}$ (fore, mid and aft chord), in Figure 23, where the ELBM solution is compared again with the experimental results and the simulation of the Euler solver [37]. Inboard, the pressure varies only slowly with the distance from the root demonstrating the two-dimensional character of the flow until it approaches the tip of the wing. There, the most intriguing features of the three-dimensional flow are present: at section x/C=0.27, a small amount of negative lift is produced near the tip; in the next two sections, this contribution returns again positive. This further confirms the presence of the rotational flow visualized by streamlines and vorticity in Figure 21.

## 5. Conclusions and Outlook: Pruned Lattices and Entropic Guided Equilibrium Construction

We reviewed some main issues arising in the framework of the compressible lattice Boltzmann method. For any finite set of discrete velocities, there is always a limit in terms of the Mach number and the temperature range to which the compressible LBM can be applied. While we have focused on the admissible lattices in our discussion, these limitations are universal by the nature of finiteness of the velocity set. Similarly, the two-population approach using the quasi-equilibrium is universally applicable to any discrete velocity set. We have demonstrated that the entropic equilibrium construction is superior to the conventional polynomial approximations in the context of compressible LBM.

In order to extend the operation domain of a given lattice, we proposed two modifications. First, the shifted lattice allows for increasing the Mach number by formulating the ELBM in a reference frame more suitable for a problem at hand. This extension requires no change in the ELBM algorithm. Second, increasing the lattice link length extends the temperature range without an increase of the number of discrete velocities. Both of these modifications were benchmarked with a number of two-dimensional simulations: The supersonic flow field around a diamond-shaped airfoil, subsonic, transonic and supersonic flow field around the Busemann biplane, viscous supersonic flow field around a NACA0012 airfoil, interaction of vortices with a shock wave, and the Richtmyer–Meshkov instability. All of the benchmarks were successfully compared, confirming the capabilities of the compressible ELBM and its extension to higher Mach numbers. We have also demonstrated a three-dimensional transonic Onera M6 wing simulation which allows for considering the ELBM approach useful for compressible flow of engineering interest.

We conclude this paper by addressing the question of how to reduce the number of populations in order to save memory and reduce the overall computational cost in the three-dimensional simulations [20]. To that end, we first suggest a pruning of the admissible lattice by dropping certain discrete velocities based on their energy shell. Second, we propose a modification of the equilibrium construction on pruned lattices by extending the guided equilibrium approach [39] which enforces accuracy on the required equilibrium moments through an extended set of constraints in the entropy minimization problem. These two techniques, lattice pruning and guided equilibrium, are considered below. The combination of both allows for decreasing by one order of magnitude the memory overhead, by three to four times the computational cost, and at the same time to increase the accuracy at high Mach numbers.

### 5.1. Lattice Pruning

Lattice pruning has already been employed in the past to reduce the number of velocities of a given lattice; as an example, the D3Q15 and the D3Q19 lattices are prunes of the $D3Q3^{3}$ lattice. A systematic theory of pruning of higher-order admissible lattices was developed in [28]; in particular, the D3Q41 lattice was derived from from the $D3Q5^{3}$ model [14,15]. Here, the pruning procedure is further extended to the $D3Q7^{3}$ lattice employed for the simulation of compressible flows. Elimination of discrete velocities from a given lattice becomes simple when symmetry considerations are taken into account. If one wants to discard an arbitrary discrete velocity (i,j,k), in order to maintain symmetry along the *x*-direction, and then to conserve the *x*-momentum, the discrete velocity (−i,j,k) also needs to be discarded. Similar considerations apply in the *y*- and *z*-directions. At this point, since (i,−j,k) have also been discarded, *x*- and *z*- reflections (−i,−j,k) and (i,−j,−k) need to be discarded. This procedure is further applied until, at the end, all discrete velocities (±i,±j,±k) are discarded. Equivalently, populations belonging to the same energy shell $\epsilon =i^{2}+j^{2}+k^{2}$ and within the same velocity shell η=|i|+|j|+|k| can be discarded. For the $D3Q7^{3}$ lattice, the number of energy shells is 19, while the number of velocity shells is 20. These are given in Table 6.

**Figure 24:**
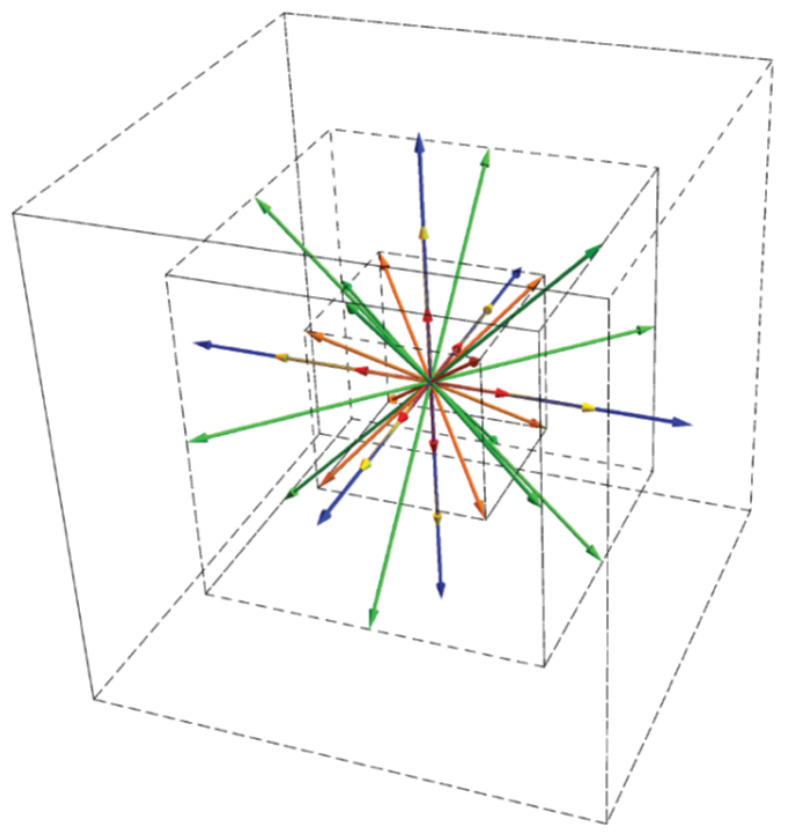
Visualization of the D3Q39 lattice. Red: ϵ=1, orange: ϵ=3, yellow: ϵ=4, green: ϵ=8, blue: ϵ=9.

A large number of combinations can be chosen in order to discard velocity and energy shells. Among others, the lattice composed by the velocity and energy shells presented in Table 7 is here considered as an example, with a total number of discrete velocities n=39. The D3Q39 lattice is visualized in Figure 24. This number of discrete velocities allows for decreasing by at least one order of magnitude the memory overhead of the computations and, in case the numerical equilibrium of Section 2.4 would be employed, to also gain one order of magnitude in the computational cost. However, the already restricted accuracy of the $DdQ7^{d}$ lattice would further degrade with discarding the discrete velocities. A modification of the entropic equilibrium construction is thus required in order to enforce the pertinent equilibrium moments to be recovered correctly. To this end, a modified version of the entropic guided equilibrium is presented in the next section.

### 5.2. Entropic Guided Equilibrium

In order to remove the aforementioned errors in the pertinent equilibrium moments, the method of guided equilibrium [39,40,41] is here employed. As for the equilibrium derivation of Section 2.4.1, an entropy function, which will be defined later, is minimized under constraints of local conservation laws of mass, momentum and energy, and, additionally, under the conditions some pertinent moments are of the Maxwell–Boltzmann form. In the present case, the conservations and enforced moments are the same as in the Grad’s thirteen moments method, i.e., the density and the momentum, the pressure tensor,

(40) $$P_{\alpha \beta }^{\mathrm{eq}}=\sum_{i=1}^{n}v_{i\alpha }v_{i\beta }f_{i}^{\mathrm{eq}}=\rho T\delta_{\alpha \beta }+\rho u_{\alpha }u_{\beta },$$

and the heat flux vector,

(41) $$q_{\alpha }^{\mathrm{eq}}=\sum_{i=1}^{n}v_{i\alpha }v_{i}^{2}f_{i}^{\mathrm{eq}}=2\rho u_{\alpha }\left(K+T\right)\delta_{\alpha \beta }+\rho u_{\alpha }u_{\beta }.$$

Note that the kinetic energy $K=\left(D/2\right)T+u^{2}/2$ is not included in the conservations since it is automatically recovered by the inclusion of the pressure tensor. We consider the entropy function of the form,

(42) $$H=\sum_{i=1}^{n}f_{i}\ln f_{i}$$

Differently from the *H* function (9), the weights are not included here for two reasons: First, the inclusion of the weights imply the equilibrium distribution to be valid only within a certain temperature range because of the positivity constraint. Second, the inclusion of the weights is not needed in the present construction since the equilibrium moments are directly enforced and do not need to be recovered by only enforcing the conservation laws. The minimization problem is expressed in terms of Lagrange multipliers, and results in

(43) $$f_{i}^{\mathrm{eq}}=\rho \exp\left(\xi +\zeta_{\alpha }v_{i\alpha }+\pi_{\alpha \beta }v_{i\alpha }v_{i\beta }+\lambda_{\alpha }v_{i\alpha }v_{i}^{2}\right),$$

where ξ(u,T), $\zeta_{\alpha }\left(u,T\right)$, $\pi_{\alpha \beta }\left(u,T\right)$, and $\lambda_{\alpha }\left(u,T\right)$ are Lagrange multipliers. As for the accurate entropic equilibrium, the closed form of $f^{\mathrm{eq}}$ is computed by inserting (43) into the specified conservation laws and equilibrium higher-order moments. In three dimensions, this results in thirteen equations for Lagrange multipliers. In addition, in this case, direct numerical evaluation of Lagrange multipliers using a rapidly converging Newton–Raphson method is employed. Clearly, inclusion of more constraints in the minimization problem leads to higher computational cost of the equilibrium. However, this is largely compensated by the reduction in the computational cost related to the lattice, and, with regard to simulations with large number of CPUs or with grid refinement [42], the advantage becomes even larger. By measuring the computational time of simulations, it has been found that the gain is of the order of 3 to 4 times with respect to the original compressible model in three dimensions.

**Figure 25:**
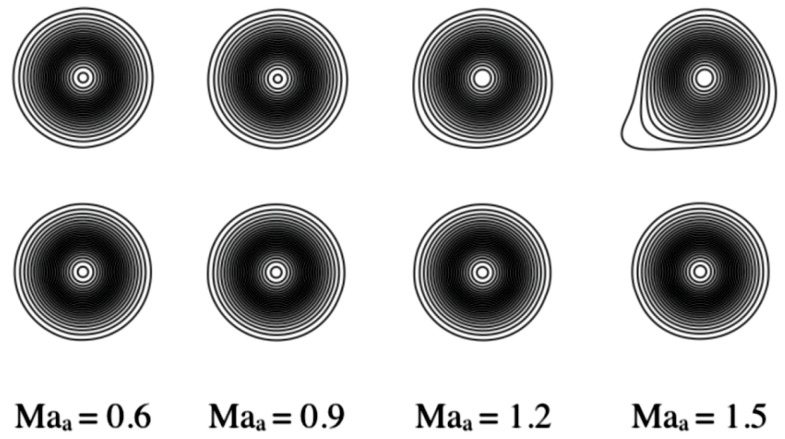
Vortex advection comparison at different advection Mach number. Top row: D3Q343 lattice with the standard entropic equilibrium. Bottom row: D3Q39 lattice with the guided entropic equilibrium.

In order to show the accuracy of combining the pruned D3Q39 lattice with the guided entropic equilibrium, in Figure 25 advection of vortices at various Mach numbers is shown for the original compressible model and the present modification. The vortex Mach number is ${\mathrm{Ma}}_{v}=0.2$. Vortices are visualized by means of pressure contours. The behavior of the vortex in Figure 25 is similar to the one of Figure 7 where the symmetric lattice was compared to the shifted (U=1) lattice: for relatively low Mach numbers, from ${\mathrm{Ma}}_{a}=0.6$ to ${\mathrm{Ma}}_{a}=0.9$, both models behave similarly, with the vortex advected correctly. Starting from a Mach number of about ${\mathrm{Ma}}_{a}=1.2$, the vortex for the case of the original compressible model starts to slightly deform; this is even more visible for the higher Mach number ${\mathrm{Ma}}_{a}=1.5$. Differently, the vortex in the case of the guided equilibrium model is not deformed, and it is advected correctly until a Mach number of around ${\mathrm{Ma}}_{a}=1.5$. The improvements brought about by the guided equilibrium on pruned lattices are evident: the reduction of the computational cost by a factor of 3–4 together with the much lower memory overhead and higher accuracy at higher Mach numbers. This renders the present optimization very promising, allowing for tackling more complex and computationally demanding simulations.

## Figures and Tables

**Table 1:** One-dimensional lattices with odd number of velocities *n*. In order: lattice velocities set $V_{n}$, minimal temperature $T_{min}$, maximal temperature $T_{max}$, reference temperature $T_{0}$, minimal reduced temperature $\theta_{min}$, maximal reduced temperature $\theta_{max}$ and temperature ratio $T_{max}/T_{min}$.

*n*	$V_{n}$	$T_{\min}$	$T_{\max}$	$T_{0}$	$\theta_{\min}$	$\theta_{\max}$	$\frac{T_{\max}}{T_{\min}}$
3	{0,±1}	0.0	1.0	1/3	−1	2	*∞*
5	{0,±1,±3}	1/3	3.0	$1\pm \sqrt{2/5}$	−0.09308	0.83772	9
7	{0,±1,±2,±3}	$1-\sqrt{2/5}$	$1+\sqrt{2/5}$	0.69795	−0.47340	1.33892	4.44
9	{0,±1,±2,±3,±5}	0.69795	2.88131	0.75608,2.17538	−0.07688	0.32451	4.13
11	{0,±1,±2,±3,±4,±5}	0.75608	2.17538	1.06279	−0.28859	1.04685	2.88

**Table 2:** Evaluation of the equilibrium using the polynomial and the numerical method, for three lattices *DdQ*$3^{d}$, *DdQ*$5^{d}$ and *DdQ*$7^{d}$, in two and three dimensions. Computational time is normalized by the fastest implementation, i.e., with the polynomial form on the *DdQ*$3^{d}$ lattice.

	*DdQ*$3^{d}$	*DdQ*$5^{d}$	*DdQ*$7^{d}$
2D			
Polynomial	1	4.12	8.13
Numerical	2.02	4.83	8.74
3D			
Polynomial	1	8.91	25.66
Numerical	1.58	10.83	29.77

**Table 3:** Computational time dependence on turbulent Mach number Ma${}_{t}$. The reference time $t_{0}$ is measured for a very low turbulent Mach number ${\mathrm{Ma}}_{t}≃0$.

${\mathrm{Ma}}_{t}$	0	0.1	0.2	0.3	0.4	0.5	0.6
$t/t_{0}$	1.000	1.047	1.023	1.059	1.062	1.070	1.067

**Table 4:** Temperature range comparison for the original *DdQ*$7^{d}$ lattice and its expanded version for increased temperature range.

$V_{7}$	$T_{\min}$	$T_{\max}$	$T_{0}$	$\theta_{\min}$	$\theta_{\max}$	$T_{\max}/T_{\min}$
{0,±1,±2,±3}	0.36754	1.63246	0.69795	−0.473397	1.33892	4.44158
{0,±1,±2,±4}	0.34969	2.71682	1.91103	−0.817014	0.42165	7.76922

**Table 5:** Comparison between analytical quantities and ELBM results for the diamond-shaped airfoil. We compare the pressure ratio $P_{*}/P_{\infty }$ and the Mach number ${\mathrm{Ma}}_{*}$ for the first half and the second half of the airfoil, denoted 1 and 2, respectively, and the leading edge oblique shock angle β; Each quantity is reported for both the upper and lower surfaces of the airfoil.

		$P_{1}/P_{\infty }$	$P_{2}/P_{\infty }$	$M_{1}$	$M_{2}$	β
Up	Analytical	1.1944	0.5616	2.0860	2.5693	29.4033
	ELBM	1.1951	0.5615	2.0859	2.5702	29.6203
	Error	$5.861\times 10^{-4}$	$1.781\times 10^{-4}$	$4.794\times 10^{-4}$	$3.503\times 10^{-4}$	$7.3810\times 10^{-3}$
Down	Analytical	1.6717	0.8334	1.8609	2.3077	34.7915
	ELBM	1.6708	0.8334	1.8581	2.3074	34.7091
	Error	$5.384\times 10^{-4}$	$1.780\times 10^{-4}$	$1.5046\times 10^{-3}$	$1.300\times 10^{-4}$	$2.3684\times 10^{-3}$

**Table 6:** Energy and velocity shells of the $D3Q7^{3}$ lattice.

ϵ	η	Representative Velocity	Number of Velocities
0	0	(0,0,0)	1
1	1	(±1,0,0)	6
2	2	(±1,±1,0)	12
3	3	(±1,±1,±1)	8
4	2	(±2,0,0)	6
5	3	(±2,±1,0)	24
6	4	(±2,±1,±1)	24
8	4	(±2,±2,0)	12
9	3	(±3,0,0)	6
9	5	(±2,±2,±1)	24
10	4	(±3,±1,0)	24
11	5	(±3,±1,±1)	24
12	6	(±2,±2,±2)	8
13	5	(±3,±2,0)	24
14	6	(±3,±2,±1)	48
17	7	(±3,±2,±2)	24
18	6	(±3,±3,0)	12
19	7	(±3,±3,±1)	24
22	8	(±3,±3,±2)	24
27	9	(±3,±3,±3)	8

**Table 7:** Energy and velocity shells of the D3Q39 lattice.

ϵ	η	Representative Velocity	Number of Velocities
0	0	(0,0,0)	1
1	1	(±1,0,0)	6
3	3	(±1,±1,±1)	8
4	2	(±2,0,0)	6
8	4	(±2,±2,0)	12
9	3	(±3,0,0)	6
